# Group membership prediction when known groups consist of unknown subgroups: a Monte Carlo comparison of methods

**DOI:** 10.3389/fpsyg.2014.00337

**Published:** 2014-05-20

**Authors:** W. Holmes Finch, Jocelyn H. Bolin, Ken Kelley

**Affiliations:** ^1^Department of Educational Psychology, Ball State UniversityMuncie, IN, USA; ^2^Department of Management, University of Notre DameNotre Dame, IN, USA

**Keywords:** discriminant analysis, mixture models, subgroup analysis, classification trees, generalized additive models

## Abstract

Classification using standard statistical methods such as linear discriminant analysis (LDA) or logistic regression (LR) presume knowledge of group membership prior to the development of an algorithm for prediction. However, in many real world applications members of the same nominal group, might in fact come from different subpopulations on the underlying construct. For example, individuals diagnosed with depression will not all have the same levels of this disorder, though for the purposes of LDA or LR they will be treated in the same manner. The goal of this simulation study was to examine the performance of several methods for group classification in the case where within group membership was not homogeneous. For example, suppose there are 3 known groups but within each group two unknown classes. Several approaches were compared, including LDA, LR, classification and regression trees (CART), generalized additive models (GAM), and mixture discriminant analysis (MIXDA). Results of the study indicated that CART and mixture discriminant analysis were the most effective tools for situations in which known groups were not homogeneous, whereas LDA, LR, and GAM had the highest rates of misclassification. Implications of these results for theory and practice are discussed.

## Introduction

The practice of classification of individuals or cases into groups is very common across nearly all branches of science. For example, classification procedures can be used to characterize psychiatric diagnoses (Zigler and Phillips, 1991), identify new or different species of animals (Britzke et al., 2011), identify individuals with learning disabilities (Keogh, 2005), or even classify stars (Bidelman, 1957), to name but a few relevant exemplars. Statistical methods for classification are often used to aid in determining if there exist clear categories, how many such categories, and to characterize the nature of these categories (i.e., classifications). Therefore, research into optimal methods of statistical classification is important due to its widespread use in a variety of disciplines in the natural and social sciences.

An important question in both methodological and applied research is the treatment and analysis of categories that are themselves composed of subcategories, sometimes but not always known. In the behavioral and social sciences, such subgroups within groups, or taxons (e.g., Meehl, 1992), are a very common occurrence, particularly when looking at diagnoses of mental disorders and cognitive functioning. For example, depressive episodes can be divided into monopolar depression and bipolar depression and then further subdivided into psychotic and non-psychotic episodes (e.g., Wedekind et al., 2007). Often severity of psychiatric disorders is studied in terms of (a) severity levels, (b) severity subgroups or (c) symptom-severity ratings (Helzer et al., 2006a,b). Such examples might include psychiatric diagnosis categorized from (a) low, to (b) moderate, to (c) high, or from “not present, mild, severe” (Helzer et al., 2006b; Kamphuis and Noordhof, 2009) or drug and alcohol abuse characterized categorized from (a) “abstinent from alcohol and other drugs,” (b) “used alcohol only,” (c) “used other drugs only,” and (d) “used alcohol and other drugs” (Schaefer et al., 2011). Another example is learning disabilities. A commonly diagnosed learning disability is attention deficit hyperactivity disorder (ADHD) which can be viewed in terms of levels of severity (Graziano et al., 2011a,b). Some children might be identified as suffering from mild ADHD, while others are classified as having moderate ADHD, and still others are identified with severe ADHD.

In each of these situations, the known group (i.e., depressed or non-depressed, ADHD or not ADHD) actually consists of subgroups based on an unmeasured level of severity. These subgroups, while all being classified under a common title such as depressed, in fact represent differing levels of the latent construct, such that they are truly qualitatively different from one another. Such mixtures of groups, while theoretically common, have not been heavily studied in the classification literature, particularly with respect to the performance of group prediction algorithms. Thus, the primary goal of this study was to extend previous work comparing the effectiveness of various group classification methods by examining the case where known groups consist of unknown subgroups.

This idea of combining continuous and categorical information to better express the degree to which the construct of interest is present within well-defined categories (i.e., measuring the severity of depression of individuals within the depressive population) is sometimes referred to as dimensional categories or the dimensional-categorical spectrum (Maysn et al., 2009). There is currently much debate in the area of psychiatric diagnosis on the proper way to categorize diagnoses in the new edition of the Diagnostic and Statistical Manual of Mental Disorders-IV (DSM-IV; American Psychiatric Association, 2000). Of the suggestions proposed, symptom-severity ratings, which combine diagnostic categories with a severity continuum, are being proposed as a promising direction to take. It is being argued that use of symptom-severity ratings provide a less arbitrary alternative to creation of diagnostic categories than the use of cut-points. Correspondingly, examination of methods that can help untangle these issues are important and needed to help test and understand existing and proposed frameworks of diagnosis.

In terms of analysis of data with dimensional categories, a few studies have suggested strategies or optimal statistical approaches. Widiger (1992) provides a review of techniques for identifying categories, dimensions, or dimensions within categories. Specifically, he discussed a variety of taxometric techniques including factor analysis, cluster analysis, maximum covariation analysis, and admixture analysis. Though strengths and weaknesses of each approach were discussed, empirical results were not presented. More recent research presents results for classification of dimensional categories by use of Bayes' theorem and base rate classification techniques (Ruscio, 2009), maximum covariance, and *k*-means cluster analysis (Beauchaine and Beauchaine, 2002).

The current research takes a different approach. Instead of considering dimensional categories as known, they are conceptualized as latent classes within known groups. For example, individuals can be diagnosed into “depressed” and “not depressed” groups based upon clinical judgment. Within these fairly broad categories, a symptom severity dimension can be thought of as a latent variable consisting of varying levels of severity. For example, an individual who has not been diagnosed with depression might in reality suffer from slight depression that is simply too weak to be identified by a clinician using standard tools for such diagnosis. In the same way, two individuals who have been diagnosed as depressed might differ in terms of their level of severity so that one suffers from relatively more severe depression while the other has a more mild case. When these latent categories exist within larger known groups, the process of classification of the known groups becomes more complicated because the known groups no longer represent homogeneous categories. Muthen (2006) presents an analysis of dimensional categories, advocating a latent variable mixture approach as a promising direction for analysis. Within the context of educational measurement and psychometrics, cognitive diagnostic models have proven to be useful for identifying latent classes within the population, while allowing for differential skill patterns within each of these classes. Within the data mining and clustering literature, there have also been advancements involving the use of k-means clustering to identify subgroups having commons sets of skills (e.g., Nugent et al., 2010), fuzzy clustering allowing for subgroup overlap (Ahmed et al., 2002), and copula based clustering algorithms for identifying clusters using mixtures of canonical variate structures (Rey and Roth, 2012). However, the existing literature has a dearth of research in the classification of known groups in the presence of latent subgroups. However, there is a real need for the investigation of appropriate methods for addressing unknown heterogeneity in known groups.

In this study we compared methods for classifying individuals into known groups when latent subgroups existed within those groups, making the known groups heterogeneous. Traditional methods for classification were included [logistic regression (LR), and linear discriminant analysis (LDA)] as were newer and more sophisticated methods [classification and regression trees (CART), generalized additive models (GAM)]. Additionally, we included a mixture approach to discriminant analysis (MIXDA). Next, we present descriptions of each method we investigate and go on to describe the study itself, present the results of our Monte Carlo simulation study, and then provide a discussion of the results of the study. We hope this study helps to push the field toward a deeper understanding of the nature of within group heterogeneity when known groups themselves consist of unknown subgroups.

## Summary of methods considered

In this section we provide a brief summary of each of the methods we evaluate. References are provided for the interest reader who would like a more detailed description of one or more of these techniques.

### Linear discrminant analysis (LDA)

Linear discriminant analysis (e.g., Mardia et al., 1979; Huberty and Olejnik, 2006) is a statistical technique that identifies the linear combination of predictor variables that maximizes the multivariate distance between groups. Based on this combination of predictors, and using a prior probability for group membership or estimating it from the data, the posterior probability of group membership is then computed for each individual in the sample and they are in turn placed in the group for which their posterior probability is highest. LDA assumes equal group variances and uses ordinary least squares for estimation.

### Logistic regression (LR)

As with LDA, LR (e.g., Hosmer and Lemeshow, 2000; Agresti, 2002) uses a linear equation involving a set of predictor variables, in this case with the log of the odds (i.e., logit) of being in one group vs. the other as the outcome. Unlike LDA, LR does not assume equal group variances and estimates parameter values using maximum likelihood.

### Classification and regression trees (CART)

The overall goal of CART is to group subjects into maximally homogeneous terminal nodes based on the outcome variable (e.g., Williams et al., 1999). The actual method involves the iterative partitioning of individuals in the sample into increasingly homogeneous groups organized around the outcome variable, based upon the set of predictors (Breiman et al., 1984). This division of individuals continues until a predefined level of homogeneity based on group membership of the outcome variable has been attained. It should be noted that, while not included in the current work due to a desire to keep the dimensions of this study manageable, there exist variations to CART that have proven to be effective classifiers as well, including the RIPPER rule learner JRIP (Witten and Frank, 2000) and the J48/C4.5 recursive partitioning model (Quinlan, 1993; Kohavi, 1995). Future research should examine the performance of these methods under conditions similar to those included in this study.

While CART has proven to be a very effective classification tool (e.g., Holden et al., 2011), it does have some weaknesses that must be considered in its use. For example, CART has a tendency to base the prediction tree on predictor variables with more distinct values over variables with fewer values, regardless of the actual differences among subjects on these (Hothorn et al., 2006). In addition, although all of the methods studied here are prone to overfitting the training data for small samples, there is evidence that this is particularly a problem for CART, making the tree less generalizable than might be desired (Berk, 2008).

### Generalized additive models (GAM)

GAM is based on combinations of smoothing functions, such as cubic splines and kernel smoothers, to predict a response variable, which can be either continuous or categorical in nature. In the case of a dichotomous outcome variable, the actual response is the logit, as with LR. The smoothing functions to be used are selected individually for each predictor so as to minimize a penalized sum of squares function, with the most common one being the cubic spline (Simonoff, 1996), which was used in the current study. As with CART, GAM also suffers from the potential problem of overfitting the model to the training data. Therefore, it is recommended that the number of smoothing parameters be kept relatively small, and that cross-validation be used to ensure that the resulting model is generalizable to other samples from the target population (Hastie and Tibshirani, 1990; Wood, 2006). Indeed, assessing the performance of all of the methods included in this study using a cross-validation sample would certainly be warranted. Prior literature has shown that CART and GAM are particularly prone to overfitting so that the use of cross-validation sample is especially important for them.

### Mixture discriminant analysis (MIXDA)

MIXDA is a variant of discriminant analysis, in which membership in each group is modeled as a mixture of Gaussian distributions, rather than a single homogeneous distribution as is the case with LDA (e.g., Hastie and Tibshirani, 1996). The MIXDA model represents each observed group by its centroid (like LDA), but also allows latent classes to exist within each known group. In other words, existing groups (e.g., diagnosed depressives and non-clinicals) can themselves contain unobserved groups of individuals. Thus, unlike LDA, MIXDA models predict group membership as a function of a mixture rather than a homogeneous distribution of the predictors. MIXDA typically relies upon the expectation maximization (EM) algorithm (Dempster et al., 1977) to estimate the model parameters including subgroup means, common or group specific variance, the within group mixing proportions, and the between group mixing proportions, all of which are obtained from the training data.

Although currently less well known in the social and behavioral sciences, MIXDA is being used with success in other fields, particularly biology (Schmid, 2009), wildlife studies (Britzke et al., 2011), and computer science (Kleinsmith et al., 2006). For example, Britzke et al. (2011) did a comparison of classification techniques for the acoustic identification of bats. Of the techniques Britzke et al. (2011) studied, MIXDA was found to produce the highest classification accuracy. Similarly, Schmid (2009) compared classification techniques for single-cell differentiation and found MIXDA to exhibit high prediction accuracy as well as provide useful procedures for visualization of the data. MIXDA has also been found to be of particular use when predictors used are non-normal (Rausch and Kelley, 2009), and when attempting to classify relatively small groups when other groups in the sample are much larger (Rausch and Kelley, 2009; Holden et al., 2011).

## Goals of the current study

The purpose of the current simulation study was to examine the performance of the previously described methods of classification when the known groups were comprised of unknown latent classes. There is ample evidence in the social science literature cited previously that such situations are fairly common in practice. Yet, very little research has been published in the methodological literature to examine how well (or poorly) these prediction algorithms might work in such situations. Based upon prior research, and the ways in which these algorithms work, we hypothesize that MIXDA should perform relatively well when subgroups exist within the larger known groups. In addition, CART and GAM have consistently demonstrated high levels of prediction accuracy across a number of simulated conditions (Grassi et al., 2001; Holden et al., 2011), leading us to believe that these methods should perform relatively well in the subgroup case as well.

## Methods

A Monte Carlo simulation study using R (version 2.13.0) software was conducted to assess the ability of the group prediction methods to correctly classify observations from known groups into the correct group from which they originated. The outcomes of interest used to judge the effectiveness of the models were the proportion of incorrectly classified individuals (i.e., misclassified) both overall, and for each group for both training data and a cross-validation (CV) sample of the same size and from the same population as the training data. The overall misclassification rate was calculated as the number of individuals predicted to be in an incorrect group divided by the total sample size. The by-group misclassification rate was the number of individuals for a given group incorrectly classified into another group, divided by the total number of individuals in the group. Five multivariate normal predictor variables were simulated for each condition, with population correlations among them drawn from the correlation matrix of the first 5 subscales of the Wechsler Adult Intelligence Scale-III (WAIS-III) reported in Waller and Jones (2009). This population level correlation matrix appears in Table 1. This correlation structure was selected because it represents what has been seen in practice in an area in which prediction methods are frequently used, the social sciences. In addition, the correlations represent a range of values seen in the literature. Several factors were manipulated in the study to assess their impact on the performance of these methods. Each of the conditions in the simulation were replicated 1000 times for the results.

**Table 1:** **Correlation matrix used in simulating predictor variable values**.

**Correlation matrix for simulated predictor variables**					
	**X1**	**X2**	**X3**	**X4**	**X5**
X1	1	0.76	0.58	0.43	0.39
X2	0.76	1	0.57	0.36	0.49
X3	0.58	0.57	1	0.45	0.74
X4	0.43	0.36	0.45	1	0.69
X5	0.39	0.49	0.74	0.69	1

### Number of known groups

Data were simulated for two and three groups. This allowed for an examination of the simplest case of multiple groups, as well as for a more complicated scenario.

### Group differences

Groups were simulated to differ on the observed variables using population standardized mean differences of 0.2, 0.5, and 0.8 (population standard deviations were 1 in all cases), corresponding to Cohen's widely used characterization of “small,” “medium,” and “large” differences for the standardize mean difference effect size measure (Cohen, 1988). In the population, the standardized mean difference is defined as

$$\delta =\frac{\mu_{1}-\mu_{2}}{\sigma },$$

where μ_*j*_ is the population mean from the *j*th group and σ is the population standard deviation assumed equal across groups, whereas the estimated standardized mean difference is

$$d=\frac{X_{1}-X_{2}}{s_{pooled}}$$

in a sample, with the sample estimates used for their population analogs in the previous equation. This approach was employed for two reasons. First, whereas multivariate mean difference effect sizes do exist, there is not agreement on which is most appropriate nor are there guidelines for interpreting their magnitudes (Kim and Olejnik, 2005). Second, the manipulation of degree of overlap among subgroups was best controlled using univariate effect sizes, as values for each variable could be changed to create a known degree of difference in group means. The actual method to do so will be described below.

### Sample size and sample size ratio

For the three groups case, whose results are featured in the Results section below, total sample size was 150, 300, or 750 to represent what some might consider small, medium, and large datasets that might be used in practice. (In the two groups case, the total sample sizes were 100, 200, and 500). Justification for this statement is provided via a review of the applied literature using group prediction methods cited in the Educational Resources Information Center database (ERIC) (July 13, 2011) revealing that these sample sizes were typical of those reported in practice. Group size ratios were simulated to be either equal, or unequal. In the unequal scenario for two groups, the ratio was 75/25, while with three groups the unequal ratio was 60/20/20. Previous research (e.g., Holden and Kelley, 2010; Holden et al., 2011) found that unequal group sizes had an impact on performance of group prediction methods, and therefore was included in the current study.

### Subgroup separation

Within each of the known groups, two subgroups were simulated to diverge from one another in terms of their population standardized means. This represents the situation in which known groups consist of unknown subgroups that the researcher might believe exists, but for which they are unsure, thus addressing a primary research question of this study. These population subgroup mean differences were set at each of 5 conditions: 0, 0.05, 0.10, 0.15, and 0.20, with the within-group population standard deviation being 1 for all groups. The subgroup mean difference of 0 corresponds to a control condition in which no subgroups are present in the data. All of the subgroups were separated to the same degree in the population within each of the known groups, and within each known group the same subgroup structure was simulated to be present.

As an example of how the data were simulated, consider the two-group situation in which the known group difference is δ = 0.8 and the subgroup separation is δ = 0.05. The first known group consisted of two subgroups, one with a population mean of 0 and population standard deviation of 1, and the other with a population mean of 0.05 and population standard deviation of 1. The second known group included two subgroups with population mean of 0.8 and population standard deviation of 1, and population mean of 0.75 and population standard deviation of 1, respectively. Note that when the known groups were separated by the smallest population mean difference (0.2), the population subgroup separation condition of 0.2 represents complete overlap of the two known groups. This scenario was included in the study purposefully in order to investigate the performance of the methods when the two known groups actually consist of completely overlapping subgroups. This condition was included in order that we address perhaps the most extreme case of group overlap. Finally, 0 subgroup separation represents the situation where no subgroups are present within the known groups, and as such serves as a control condition in this simulation study.

### Subgroup ratio

The subgroups were simulated to be of equal size or with ratio of 75/25. Prior research (e.g., Holden et al., 2011) has demonstrated that the unequal group ratios have an impact on the performance of group classification methods such as those examined in this study. In the 75/25 condition, the larger subgroup was the one furthest from the other known group. For example, consider known group 1 consisting of subgroups A and B, with means of 0 and 0.1, and known group 2 consisting of subgroups C and D, with means of 0.7 and 0.8. In the unequal subgroups condition, subgroup A would be the larger in known group 1, and subgroup D would be the largest in known group 2.

### Prediction methods

Prediction of group membership was done using the methods discussed: (a) LDA, (b) LR, (c) MIXDA, (d) CART, and (e) GAM. LDA and LR were included because they are the most popular group prediction methods, based on the ERIC database search mentioned above. For LDA, the model prior probabilities were taken from the data, which is recommended in general practice (Huberty and Olejnik, 2006). CART and GAM were included because they have been shown in previous research (e.g., Holden et al., 2011) to provide very accurate group predictions under a variety of conditions. For CART the deviance criterion was used as the splitting rule, with a minimum deviance to split value of 0.01, and a minimum terminal node size of 10 observations. In other words, CART would stop splitting if the reduction in deviance was not at least 0.01, or if a resulting node would include fewer than 10 observations. Both of these conditions are recommended by Williams et al. (1999). For GAM, the value of epsilon was set at 0.0000007, with a maximum of 30 iterations for the estimation algorithm. As with CART, these settings are recommended in the literature (Wood, 2006). MIXDA was included because it was explicitly designed to find subgroups within broader known groups and to use that information to yield more accurate predictions of the primary groups of interest (Hastie et al., 2001). However, although developed with this purpose in mind, it has not, to our knowledge, been extensively studied in a Monte Carlo simulation in order to determine if it is in fact more accurate when known groups contain subgroups. With respect to the settings for MIXDA, the convergence tolerance for the EM algorithm was set to 0.00005, and the maximum number of iterations was 100.

## Results

The results of the simulation study are presented in the following sections. Because results for the two and three groups cases were very comparable, only the three groups results are presented here, in order to keep the scope of results manageable. Two groups results are available upon request from the authors. First, overall rates of misclassification are examined, followed by individual group misclassification. The results are presented for the cross-validation sample in all cases. The misclassification rates for the training sample followed an identical pattern to the cross-validation results presented below, with the only difference being that they were 0.04 lower, on average; i.e., the results for the training sample were somewhat more accurate than for the cross-validation sample. Finally, given the focus of the study on the impact of the presence of unknown subgroups within known groups, the results will be framed in the context of the overlap among the subgroups.

### Overall misclassification

Focusing first on prediction method differences across all conditions, Table 2 contains misclassification rates for each method by the degree of overlap among the subgroups. Across the study conditions CART exhibited the lowest misclassification rates, while LDA had the highest. MIXDA had the second lowest rates of overall misclassification, while GAM performed similarly to LDA, and LR had rates that fell in the middle. All of the methods experienced an increase in overall misclassification as the degree of overlap increased (i.e., group separation decreased). In the control condition (overlap = 0), each method yielded the lowest rates of misclassification, except MIXDA, for which the overlap = 0.05 actually had slightly lower misclassification than in the no overlap case. In other words, MIXDA was slightly more accurate at classification when there were subgroups within the main groups, which is not surprising given that it was designed to model the presence of such subgroups. All of the methods had the highest overall misclassification rates for an overlap of 0.20 (the smallest group separation studied).

**Table 2:** **Overall misclassification rates by method and subgroup overlap**.

**Overlap**	**LDA**	**LR**	**MIXDA**	**CART**	**GAM**
0.00	0.510	0.446	0.412	0.321	0.498
0.05	0.522	0.460	0.408	0.328	0.512
0.10	0.529	0.466	0.425	0.349	0.521
0.15	0.542	0.472	0.440	0.363	0.529
0.20	0.559	0.480	0.481	0.382	0.538

Table 3 includes the overall misclassification rates for each prediction method by the degree of subgroup overlap (overlap), total sample size (N), known group sample size ratio (Nratio), subgroup ratio (Sratio), and distance between known group means (D). The focus here is on the extent to which the impact of overlap on overall misclassification rates was affected by the other manipulated variables in the study. With respect to sample size, LDA uniformly yielded lower overall misclassification rates with larger samples, regardless of the degree of overlap. On the other hand, both LR and GAM yielded the highest misclassification rates for *N* = 300, and the lowest misclassification rates for total sample size of 750. Finally, both CART and MIXDA had increasing error rates with increasing overall sample size values, for each level of subgroup overlap. In terms of the known group sample size ratio (Nratio), all of the prediction methods except MIXDA yielded lower values in the 75/25 case, as opposed to the equal group size condition. However, just the opposite result was seen for MIXDA, in which known group size inequality resulted in lower overall misclassification.

**Table 3:** **Overall misclassification rates by method, degree of subgroup overlap (overlap), sample size (N), known group sample size ratio (Nratio), subgroup sample size ratio (Sratio), and difference in known group means (D)**.

	**Overlap**	**LDA**	**LR**	**MIXDA**	**CART**	**GAM**
***N***						
150	0.00	0.548	0.469	0.369	0.268	0.511
	0.05	0.563	0.477	0.323	0.248	0.513
	0.10	0.571	0.482	0.327	0.266	0.523
	0.15	0.588	0.490	0.366	0.276	0.534
	0.20	0.595	0.498	0.408	0.306	0.546
300	0.00	0.514	0.453	0.386	0.294	0.511
	0.05	0.546	0.484	0.419	0.333	0.548
	0.10	0.554	0.492	0.454	0.365	0.559
	0.15	0.568	0.499	0.446	0.384	0.569
	0.20	0.585	0.505	0.506	0.403	0.576
750	0.00	0.469	0.416	0.481	0.401	0.471
	0.05	0.458	0.418	0.483	0.403	0.475
	0.10	0.463	0.424	0.495	0.417	0.481
	0.15	0.470	0.429	0.508	0.429	0.485
	0.20	0.498	0.436	0.528	0.438	0.490
**NRATIO**						
Equal	0.00	0.583	0.501	0.403	0.350	0.529
	0.05	0.589	0.512	0.395	0.355	0.539
	0.10	0.600	0.519	0.416	0.384	0.547
	0.15	0.619	0.527	0.426	0.404	0.556
	0.20	0.644	0.537	0.476	0.431	0.565
75/25	0.00	0.365	0.335	0.430	0.262	0.434
	0.05	0.388	0.356	0.435	0.275	0.458
	0.10	0.388	0.360	0.444	0.281	0.469
	0.15	0.389	0.363	0.468	0.282	0.476
	0.20	0.391	0.366	0.490	0.285	0.483
**SRATIO**						
Equal	0.00	0.478	0.421	0.371	0.287	0.480
	0.05	0.489	0.434	0.357	0.290	0.492
	0.10	0.494	0.439	0.379	0.317	0.501
	0.15	0.504	0.444	0.396	0.335	0.510
	0.20	0.517	0.451	0.454	0.360	0.518
75/25	0.00	0.574	0.495	0.494	0.389	0.533
	0.05	0.588	0.512	0.511	0.404	0.553
	0.10	0.599	0.520	0.518	0.413	0.560
	0.15	0.619	0.529	0.527	0.420	0.568
	0.20	0.644	0.538	0.534	0.427	0.576
***D***						
0.2	0.00	0.607	0.537	0.494	0.392	0.606
	0.05	0.597	0.541	0.506	0.408	0.615
	0.10	0.607	0.545	0.528	0.445	0.620
	0.15	0.636	0.551	0.540	0.466	0.623
	0.20	0.674	0.557	0.625	0.499	0.624
0.5	0.00	0.499	0.445	0.432	0.328	0.505
	0.05	0.518	0.459	0.401	0.318	0.515
	0.10	0.524	0.467	0.412	0.332	0.526
	0.15	0.530	0.475	0.433	0.342	0.539
	0.20	0.536	0.481	0.449	0.358	0.550
0.8	0.00	0.426	0.355	0.311	0.244	0.381
	0.05	0.451	0.380	0.319	0.258	0.406
	0.10	0.457	0.386	0.335	0.272	0.416
	0.15	0.461	0.391	0.347	0.281	0.427
	0.20	0.469	0.401	0.369	0.291	0.439
	Min	0.23	0.20	0.01	0.15	0.26
	Max	0.83	0.63	0.71	0.67	0.71
	Median	0.52	0.46	0.44	0.32	0.54
	Mean	0.53	0.46	0.43	0.35	0.52
	IQR	0.15	0.12	0.16	0.19	0.23

**Figure 1:**
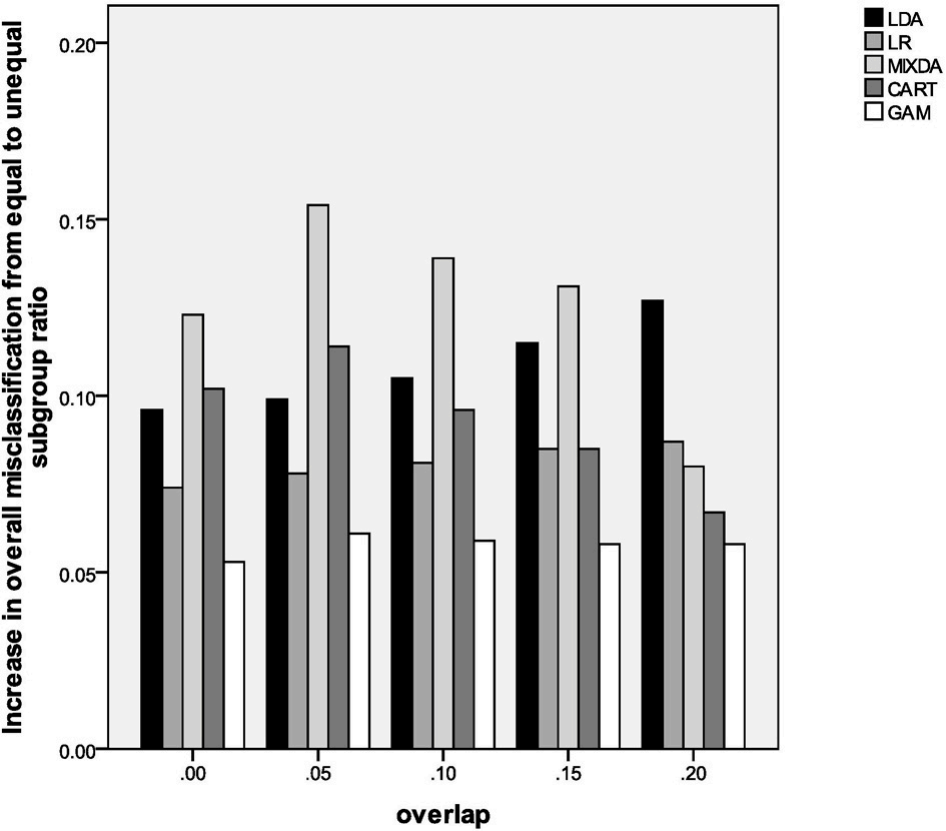
**Increase in overall misclassification rate from equal subgroup ratio to 75/25 ratio, by method and degree of subgroup overlap**.

In contrast to the impact of known group sample size ratio, the presence of unequal subgroup sizes (Sratio) yielded inflated misclassification rates for all methods, when compared to the equal subgroup size case. The impact of subgroup inequality was particularly notable for MIXDA, as can be seen in Figure 1. This figure presents the increase in overall misclassification rate from the equal subgroup to 75/25 subgroup ratio conditions by degree of subgroup overlap and method of classification. Thus, in the 0.0, 0.05, 0.10, and 0.15 overlap conditions MIXDA yielded the greatest increases in overall misclassification when going from equal to unequal subgroup sizes. CART had the second greatest increase in misclassification for overlap of 0 and 0.05, while LDA had the second highest rates of misclassification increase for overlap of 0.10 and 0.15, and the highest such rates for overlap of 0.20. On the other hand, GAM consistently had the smallest increase in the misclassification rates from the equal to 75/25 condition. In other words, it was least sensitive to differences in subgroup sizes. Finally, an examination of results in Table 2 show that as the difference in means for the known groups increased, the misclassification rates for all methods decreased, across levels of subgroup overlap.

At the bottom of Table 3 are included marginal descriptive statistics for each method, including the overall mean proportion of misclassified cases, as well as the median, minimum, maximum, and interquartile range (IQR). These values help to shed further light on the relative performance of the classification methods considered here. For example, across all conditions, the mean and median of CART were the lowest, further reinforcing the finding that it is the most accurate method considered here, while GAM and LDA demonstrated themselves to be the least accurate, with the misclassification rates of LR and MIXDA lying in between. However, the single lowest rate belonged to MIXDA, while the single highest was for LDA. Finally, with regard to the IQR, which is a reflection of variation in misclassification rates, LR had the lowest value, while GAM had the highest.

### Individual group misclassification

In addition to the overall misclassification rates, we also examined the individual group misclassification rates. As a reminder, these were calculated as the number of individuals in a group who were misclassified divided by the total number in the group. In the equal known group size condition, results for the first and third groups were very similar, while results for the second and third (the two smaller) groups were very similar for the unequal group size case. Therefore, in order to simplify presentation of results as much as possible, only results for groups 1 and 2 are included here.

Table 4 includes the group misclassification rates by method and degree of overlap. For all methods except GAM, rates for group 1 were lower than those for group 2. In other words, misclassification was more likely to occur for the middle group than either of the end groups (remembering that group 3 showed very similar patterns to group 1). In addition, from this table it is clear that misclassification for the middle group (2) under LDA increased as the degree of subgroup overlap increased. For the other methods, while there were some differences in misclassification for different levels of overlap, there was no clear pattern associating changes in degree of overlap with misclassification rates. The differential in misclassification rates for groups 1 and 2 was smallest for MIXDA, and greatest for GAM, although as noted previously, rates for group 1 were higher for group 2 with GAM, contrary to the results for the other methods.

**Table 4:** **By group misclassification rates by method and subgroup overlap (overlap)**.

**Overlap**	**LDA1**	**LDA2**	**LR1**	**LR2**	**MIXDA1**	**MIXDA2**	**CART1**	**CART2**	**GAM1**	**GAM2**
0.00	0.382	0.579	0.330	0.603	0.420	0.531	0.299	0.409	0.754	0.206
0.05	0.353	0.615	0.310	0.646	0.428	0.477	0.274	0.413	0.748	0.208
0.10	0.361	0.665	0.308	0.638	0.447	0.505	0.288	0.440	0.727	0.245
0.15	0.392	0.701	0.325	0.668	0.440	0.538	0.312	0.444	0.709	0.264
0.20	0.417	0.689	0.348	0.602	0.437	0.500	0.314	0.410	0.732	0.254

In order to investigate further the impact of overlap in conjunction with other factors manipulated in this study, we refer to Table 5. Several results presented in this table simply mirror those that were in evidence for the overall misclassification rates, and will therefore not be described in detail here. Namely, the individual group rates generally declined concomitantly with greater difference in the known group means, and were generally higher when the subgroups were of unequal sizes. Of particular interest with respect to group specific misclassification rates was the impact of unequal group sizes (Nratio). When interpreting these results, it is important to remember that in the unequal group size condition, group 1 was the larger, and groups 2 and 3 were smaller, and of equal size. For LDA, LR, and CART the presence of unequal known group sizes resulted in lower misclassification rates for all groups, regardless of the degree of overlap. For MIXDA the misclassification rates switched in a sense, with group 1 having lower rates in the equal group size condition, and group 2 having lower rates in the unequal group size condition. Finally, contrary to what was in evidence for the other methods, GAM exhibited higher misclassification rates for both groups in the unequal known group condition.

**Table 5:** **By group misclassification rates by method, degree of subgroup overlap (overlap), sample size (N), known group sample size ratio (Nratio), subgroup sample size ratio (Sratio), and difference in known group means (D)**.

	**Overlap**	**LDA1**	**LDA2**	**LR1**	**LR2**	**MIXDA1**	**MIXDA2**	**CART1**	**CART2**	**GAM1**	**GAM2**
***N***											
150	0.00	0.338	0.582	0.290	0.616	0.542	0.538	0.285	0.322	0.768	0.204
	0.05	0.388	0.651	0.314	0.660	0.416	0.364	0.249	0.259	0.728	0.191
	0.10	0.407	0.703	0.314	0.669	0.480	0.426	0.286	0.319	0.763	0.207
	0.15	0.414	0.721	0.318	0.670	0.511	0.408	0.279	0.270	0.756	0.225
	0.20	0.413	0.726	0.338	0.631	0.565	0.378	0.287	0.273	0.705	0.258
300	0.00	0.333	0.577	0.301	0.624	0.421	0.474	0.239	0.375	0.783	0.189
	0.05	0.354	0.634	0.317	0.677	0.464	0.505	0.257	0.417	0.756	0.210
	0.10	0.370	0.676	0.325	0.687	0.509	0.555	0.285	0.471	0.754	0.229
	0.15	0.399	0.683	0.327	0.696	0.434	0.580	0.302	0.488	0.732	0.256
	0.20	0.464	0.702	0.385	0.655	0.427	0.597	0.341	0.474	0.752	0.253
750	0.00	0.470	0.578	0.394	0.572	0.313	0.581	0.373	0.520	0.713	0.226
	0.05	0.317	0.561	0.299	0.600	0.403	0.562	0.315	0.562	0.760	0.224
	0.10	0.303	0.615	0.284	0.551	0.344	0.530	0.293	0.525	0.661	0.302
	0.15	0.363	0.700	0.330	0.635	0.378	0.621	0.356	0.569	0.635	0.311
	0.20	0.359	0.631	0.313	0.501	0.303	0.514	0.312	0.484	0.736	0.252
**NRATIO**											
Equal	0.00	0.485	0.715	0.417	0.740	0.341	0.685	0.328	0.511	0.693	0.195
	0.05	0.440	0.726	0.386	0.774	0.304	0.583	0.278	0.503	0.706	0.166
	0.10	0.480	0.754	0.405	0.792	0.352	0.647	0.323	0.555	0.716	0.179
	0.15	0.509	0.763	0.414	0.798	0.358	0.665	0.345	0.538	0.705	0.198
	0.20	0.586	0.783	0.482	0.743	0.327	0.657	0.367	0.516	0.736	0.166
75/25	0.00	0.189	0.324	0.168	0.347	0.569	0.241	0.246	0.219	0.868	0.227
	0.05	0.181	0.394	0.158	0.388	0.674	0.264	0.266	0.232	0.831	0.294
	0.10	0.149	0.507	0.137	0.363	0.617	0.254	0.225	0.235	0.747	0.364
	0.15	0.145	0.567	0.136	0.391	0.615	0.267	0.242	0.243	0.716	0.402
	0.20	0.134	0.532	0.126	0.367	0.620	0.238	0.227	0.234	0.724	0.402
**SRATIO**											
Equal	0.00	0.322	0.493	0.280	0.521	0.431	0.408	0.277	0.336	0.832	0.178
	0.05	0.310	0.561	0.271	0.583	0.447	0.333	0.252	0.332	0.784	0.201
	0.10	0.298	0.619	0.256	0.557	0.473	0.353	0.252	0.371	0.764	0.245
	0.15	0.321	0.666	0.277	0.597	0.458	0.398	0.284	0.386	0.757	0.247
	0.20	0.341	0.644	0.290	0.543	0.423	0.408	0.303	0.372	0.750	0.261
75/25	0.00	0.476	0.714	0.407	0.731	0.403	0.722	0.334	0.523	0.632	0.251
	0.05	0.440	0.725	0.388	0.771	0.388	0.766	0.317	0.574	0.674	0.224
	0.10	0.471	0.747	0.402	0.782	0.402	0.776	0.351	0.561	0.662	0.247
	0.15	0.519	0.763	0.411	0.794	0.410	0.788	0.363	0.548	0.622	0.294
	0.20	0.599	0.798	0.490	0.745	0.471	0.723	0.342	0.503	0.688	0.237
***D***											
0.2	0.00	0.548	0.746	0.423	0.728	0.533	0.670	0.386	0.482	0.759	0.308
	0.05	0.401	0.744	0.328	0.765	0.563	0.607	0.316	0.534	0.762	0.309
	0.10	0.418	0.856	0.323	0.737	0.542	0.620	0.321	0.583	0.632	0.404
	0.15	0.463	0.870	0.327	0.738	0.465	0.605	0.344	0.502	0.549	0.472
	0.20	0.575	0.903	0.420	0.572	0.478	0.575	0.386	0.461	0.489	0.535
0.5	0.00	0.333	0.557	0.311	0.614	0.413	0.514	0.280	0.408	0.838	0.137
	0.05	0.356	0.592	0.323	0.636	0.406	0.461	0.283	0.382	0.877	0.110
	0.10	0.353	0.607	0.317	0.628	0.412	0.443	0.282	0.358	0.908	0.110
	0.15	0.402	0.694	0.364	0.716	0.462	0.552	0.320	0.450	0.925	0.092
	0.20	0.378	0.659	0.340	0.663	0.460	0.525	0.310	0.441	0.948	0.103
0.8	0.00	0.278	0.441	0.261	0.464	0.316	0.414	0.237	0.338	0.642	0.194
	0.05	0.302	0.509	0.279	0.536	0.313	0.363	0.223	0.322	0.604	0.206
	0.10	0.304	0.509	0.283	0.535	0.375	0.439	0.256	0.359	0.654	0.202
	0.15	0.303	0.517	0.283	0.542	0.391	0.448	0.268	0.373	0.672	0.201
	0.20	0.321	0.535	0.295	0.560	0.375	0.406	0.256	0.330	0.700	0.180

**Figure 2:**
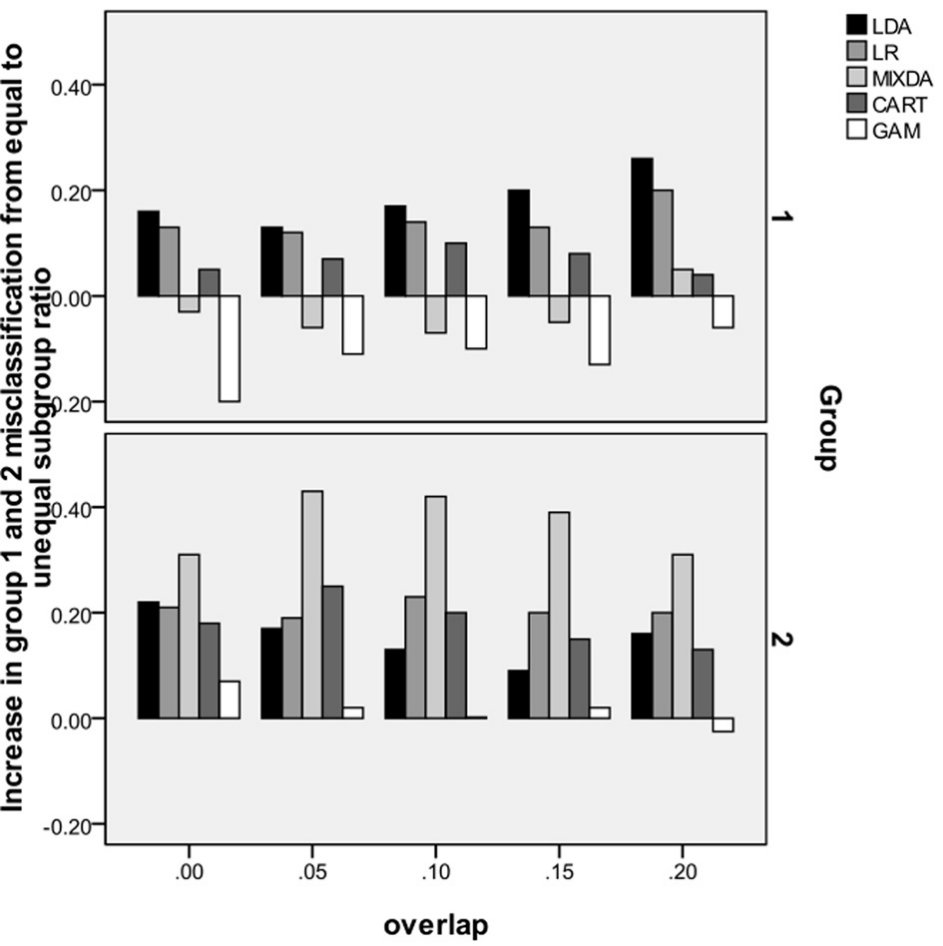
**Increase in misclassification rate from equal subgroup ratio to 75/25 ratio, by method, group and degree of subgroup overlap**.

In order to mirror results presented above for the overall misclassification rate, and further investigate the impact of subgroup size ratio on misclassification by group, we refer to Figure 2. Similar to Figure 1, along the *y*-axis is the difference between group misclassification rates for the equal and unequal subgroup conditions, calculated as misclassification rate for unequal subgroup ratio–misclassification rate for equal subgroup ratio. On the *x*-axis is the degree of subgroup overlap, and groups 1 and 2 each have their own panels. Based on the results in this figure, it appears that the impact of going from equal to unequal subgroup sizes was greatest on MIXDA for the second group. In that case, the increase in misclassification for the unequal subgroup condition compared to equal, ranged from 0.3 to 0.43. While not such a marked difference, for most of the methods studied here, the misclassification rates for both groups was higher in the unequal subgroup condition. In addition to MIXDA, this impact was greater in group 2 for LR and CART. For LDA, the difference in group 1 misclassification rates between the unequal and equal subgroup conditions increased with greater subgroup overlap. In other words, the more the subgroups overlapped with one another, the larger disparity in classification accuracy between the equal and unequal subgroup size conditions. Conversely, GAM displayed a very different pattern than the other methods for group 1 in that the misclassification in the equal subgroup size condition was actually greater than in the unequal, as evidenced by the negative values associated with the bars. Furthermore, this difference in misclassification declined with greater subgroup separation; i.e., the bars got closer to 0. With regard to group 2, there was very little difference in GAM's misclassification rates between the equal and unequal subgroup size conditions. Table 6 includes a summary of the simulation results.

**Table 6:** **Summary of simulation study results**.

**Manipulated variable**	**Result**
Method	CART had lowest misclassification rates; MIXDA had second lowest misclassification rates; LDA, GAM, and LR had highest misclassification rates
Overlap	More overlap led to higher misclassification
*N*	Larger N generally led to lower misclassification rates for LDA, GAM, and LR. Larger N led to higher misclassification rates for CART and MIXDA
Nratio	Known group size inequality led to lower misclassification rates for all methods except MIXDA
Subgroup ratio	Subgroup size inequality led to higher misclassification rates for all methods
Group separation	Greater known group separation led to lower misclassification rates for all methods

## Discussion

The goal of this study was to compare the performance of five methods for classification when known groups consist of multiple latent subgroups. Such a situation arises, for example, when individuals who have been diagnosed with depression are compared to those who are non-clinical, but within each group there exist differentiated levels of actual depression severity. While such situations are common in the applied literature (e.g., Meehl, 1992), very little methodological research has examined the performance of statistical classification tools when such subgroups within known groups are present. We hope that the current study will serve as a first step in this direction, providing both applied and methodological researchers with information on the performance of these methods for addressing known group classification when subgroups are present. The methods included in this study were selected either because they are popular in practice (LDA and LR), have been shown in prior research to be optimal in many classification situations (CART and GAM), or theoretically should be optimal for situations in which known groups consist of mixtures (MIXDA).

These results show that two of the most popular methods for classification, LDA and LR, do not perform well in the presence of known group mixtures. Indeed, LDA consistently displayed the worst results of all methods across virtually all of the conditions in our Monte Carlo simulation study. These results are not completely surprising in that prior simulation studies have shown these methods to be less accurate when no subgroups are present in the data (Holden et al., 2011), but to our knowledge it has not been demonstrated when subgroups exist within known groups. Given that LDA and LR rely on linear combinations of the predictors in order to develop a prediction equation, there is no reason to expect that their relative performance would improve when the classification is complicated by the presence of latent subgroups within the larger known groups. Indeed, it is reasonable to expect that any non-linearities introduced into the data as a result of subgroups being present in the data will not be adequately dealt with by these linear modeling methods. Nonetheless, it is important to note that they continue to perform relatively less well than alternative methods in this more complex case.

Also in keeping with prior research (e.g., Holden et al., 2011), under most of the simulated conditions CART provided the most accurate predictions, regardless of the degree of subgroup separation within the known groups. While CART has previously been demonstrated to be among the most accurate classification tools when known groups do not consist of mixtures, it was not known how well this method would work in the presence of subgroups. A potential strength of CART in many classification situations is that it is non-parametric in nature (Breiman et al., 1984). Thus, unlike the other methods studied here, which assume a specific model structure relating the predictors to the grouping variable, CART develops its prediction tree using a partitioning algorithm that, at each step in the process, identifies the division of the data that provides the most accurate classification possible. Thus, while this study was designed primarily to be an exploration of the subgroups' impact on classification accuracy, it is possible to assert a hypothesis that CART's partitioning algorithm was better able to ignore the noise created by the subgroups and more accurately identify differentiations between the known groups, than were the model based approaches, all of which rely on some linear or non-linear combination of the variables in developing a prediction equation. When the groups to be classified contain a great deal of statistical variation due to the existence of the subgroups, the resulting parameter estimates for the model based coefficients would themselves contain added noise, making them less accurate. In other words, it appears that CART's partitioning algorithm was better able to identify the differences among the broader known groups than were the models underlying the other methods.

Typically, the second best performer in this study was MIXDA, which explicitly allows for the presence of subgroups, and attempts to identify them in the development of a prediction algorithm for the known groups (Hastie and Tibshirani, 1996). Correspondingly, it was hypothesized that MIXDA would perform well in comparison to the other methods. However, results of this study revealed that CART provided more accurate group classification than MIXDA, even when the degree of separation between subgroups was relatively high. This is a new finding in the literature and shows the flexibility and robustness of CART, along with its stability across a wide variety of realistic situations encountered in practice. However, these results do indicate that MIXDA is preferable to the other model based approaches studied here when there are subgroups within the broader known groups. In addition, though not a focus of this study, it should be noted that one feature of MIXDA that is not associated with the other approaches, including CART, is the explicit identification of subgroup membership. Therefore, when using MIXDA it is possible not only to predict an individual's membership in the larger known group, but also to obtain their membership in the subgroups as well. Such information could prove to be quite important to researchers interested in learning about such subgroups, making MIXDA potentially useful in such cases. Indeed, given that in many instances MIXDA performed only slightly worse than CART, this additional information regarding subgroup membership may make the MIXDA approach preferable when researchers are interested in understanding not only known group membership, but also membership among the within group mixtures.

Finally, GAM, which had been shown to be accurate in prior studies of group classification when no subgroups were present (e.g., Holden et al., 2011), did not perform particularly well when the known groups consisted of mixtures. Indeed, in many cases it was no better than LDA, which is quite a different result from the prior work where GAM was typically among the most accurate performers. Thus, it seems clear that the noise created by the presence of subgroups in the data could lead to coefficient estimation problems in GAM that are similar to those witnessed in LDA. Furthermore, in earlier studies GAM was particularly effective when the relationship between group membership and the predictors was non-linear, which was not the case here. It remains to be seen whether the relative performance of GAM to the other methods would improve in such a case. Nevertheless, it does not seem likely that it would outperform the recursive partitioning algorithm underlying CART, given the latter's clear dominance in this study and good performance in the non-linear case with no subgroups (Holden et al., 2011).

Another consistent finding of this study was that misclassification rates for the middle group of three was nearly always higher than for either of the end groups. This result matches earlier work (Finch and Schneider, 2007), and highlights a potential problem for researchers who are particularly interested in classification for a group whose means reside in the middle of several other groups. In their work, Finch and Schneider noted that a potential problem for classifying such a middle group is that misclassification can occur in two possible ways, with individuals being misclassified into either adjacent group. However, for the more extreme groups on the ends, misclassification into the other extreme group is much less likely than misclassification into the adjacent category. And indeed, analysis of some individual simulation results demonstrated this to be the case.

In applying these results to practice, researchers must clearly be cognizant of the possibility that the known groups of interest may in turn consist of latent subgroups. The presence of such subgroups will cause difficulty in the development of an accurate classification algorithm, and the subsequent proper classification of individuals in a cross-validation sample. Indeed, the more pronounced the subgroup separation, the more problems will occur for the classification algorithms. Model based methods of classification, which rely on estimation of coefficients for the predictors, seem particularly susceptible to the noise created by these subgroups. This result has very real implications for researchers interested in using these classification methods, as they must consider the extent to which such subgroups might be present in the data. An investigation into this issue would seem to imply that the careful use of descriptive statistics and graphing techniques are recommended so as to identify the potential presence of such subgroups, in addition to being guided by theory and the literature. Furthermore, when the researcher suspects that latent subgroups may be present, he or she should consider *not* using the more familiar, but in this case less accurate, classification tools such as LDA and LR, but rather *should* select more robust methods such as CART or MIXDA. Indeed, given CART's stellar performance in prior research where known groups consisted of homogeneous sets of individuals rather than mixtures, one could argue that in conditions similar to those included in simulation studies, CART may be an optimal method for group classification in general. One exception to this recommendation, however, would be the case where the researcher is interested in the subgroups themselves, and does not view them as merely a nuisance. In such cases, MIXDA is probably preferable to CART, given its relative accuracy for group prediction and the ability to explicitly model the subgroups existing within the known groups, which CART cannot do.

## Limitations and directions for future research

The goal of this study was to compare classification accuracy of group memberships when the groups contain latent subgroups for five methods that have proven popular and/or been strong performers in previous studies. Nevertheless, not all possible classification methods were included, primarily because they have not proven optimal previously and the added complexity of what we studied did not seem conducive to their performance. Among these are neural networks and multivariate adaptive regression splines (MARS). While they were not found to be top performers in studies when known groups were homogeneous, it is not known how they would compare in the situations like those simulated here. Thus, future work should compare these methods with some of the current strong performers, such as CART and MIXDA.

In addition, the current study used three univariate effect sizes to simulate known group, and subgroup separation. These were selected because they have been used in prior research and provide a stable and well understood metric for defining group differences. At the same time, there are obviously other levels of group differences that could have been explored, and other methods for describing such difference. While we are comfortable with the methodology chosen here, we also recognize the possibility for other approaches to be used. One problem with using a multivariate measure of group separation such as the Mahalanobis distance is that there are not generally agreed upon notions of what constitutes a small or large difference. Thus, using this approach to simulating group separation would raise the difficulty of deciding on appropriate magnitudes. Future work should investigate these problems from a multivariate perspective.

Finally, future research should investigate a different set of conditions, expanding on those included here. As the study was mainly exploratory due to the lack of research on this topic, the number of conditions was kept to a reasonable number for ease of interpretation. However, now that the groundwork has been laid, there are many interesting directions that can be investigated to further our understanding of these and related topics. For example, while both 2 and 3 groups were studied here, future research could examine, perhaps, 5 groups, which lies at the upper boundary of most applied classification studies with which we are familiar. The impact of different covariance structures for both the predictor variables and the subgroups would also be of interest. In addition, future work should also include a wider range of predictor variables, and different distributions of the predictors, as well as different numbers of mixtures within the known groups.

### Conflict of interest statement

The authors declare that the research was conducted in the absence of any commercial or financial relationships that could be construed as a potential conflict of interest.

## References

[B1] Agresti A. (2002). Categorical Data Analysis. Hoboken, NJ: Wiley

[B2] Ahmed M. N. Yamany S. M. Mohamed N. Farag A. A. Moriarty T. (2002). A modified fuzzy C-means algorithm for bias field estimation and segmentation of MRI data. IEEE Trans. Med. Imaging 21, 193–199 10.1109/42.996338 11989844

[B3] American Psychiatric Association. (2000). Diagnostic and Statistical Manual IV. Washington, DC: American Psychiatric Association

[B4] Beauchaine T. P. Beauchaine R. J. (2002). A comparison of maximum covariance and k-means cluster analysis in classifying cases into known taxon groups. Psychol. Methods 7, 245–261 10.1037/1082-989X.7.2.245 12090413

[B5] Berk R. A. (2008). Statistical Learning From a Regression Perspective. New York, NY: Springer

[B6] Bidelman W. P. (1957). Spectral classifications of stars noted on Case Objective Prism Plates. II. Publ. Astron. Soc. Pac. 69, 326–332 10.1086/127079

[B7] Breiman L. Friedman J. Olshen R. A. Stone C. J. (1984). Classification and Regression Trees. Monterey, CA: Wadsworth and Brooks/Cole

[B8] Britzke E. R. Duchamp J. E. Murray K. L. Swihart R. K. Robbins L. W. (2011). Acoustic identification of bats in the eastern United States: a comparison of parametric and nonparametric methods. J. Wildl. Manage. 75, 660–667 10.1002/jwmg.68

[B9] Cohen J. (1988). Statistical Power Analysis for the Behavioral Sciences. 2nd Edn Hillsdale, NJ: Lawrence Erlbaum

[B10] Dempster A. P. Laird N. M. Rubin D. B. (1977). Maximum likelihood from incomplete data via the EM algorithm. J. R. Stat. Soc. B (Methodol.) 39, 1–38

[B11] Finch H. Schneider M. K. (2007). Classification accuracy of neural networks vs. discriminant analysis, logistic regression and classification and regression trees: three and five groups cases. Methodology 3, 47–57

[B12] Grassi M. Villani S. Marinoni A. (2001). Classification methods for the identification of “case” in epidemiological diagnosis of asthma. Eur. J. Epidemiol. 17, 19–29 11523572 10.1023/a:1010987521885

[B13] Graziano P. A. Geffken G. R. Lall A. S. (2011a). Heterogeneity in the pharmacological treatment of children with ADHD: cognitive, behavioral, and social functioning differences. J. Atten. Disord. 15, 382–391 10.1177/1087054710367772 20495162

[B14] Graziano P. A. McNamara J. P. Geffken G. R. (2011b). Severity of children's ADHD symptoms and parenting stress: a multiple mediation model of self-regulation. J. Abnorm. Child Psychol. 39, 1073–1083 10.1007/s10802-011-9528-0 21629991

[B15] Hastie T. Tibshirani R. (1990). Generalized Additive Models. Chapman and Hall 10.1177/096228029500400302 8548102

[B16] Hastie T. Tibshirani R. (1996). Discriminant analysis by Gaussian mixtures. J. R. Stat. Soc. B (Methodol.) 58, 155–176

[B17] Hastie T. Tibshirani R. Friedman J. (2001). The Elements of Statistical Learning: Data Mining, Inference, and Prediction. New York, NY: Springer 10.1007/978-0-387-21606-5

[B18] Helzer J. E. Kraemer H. C. Krueger R. F. (2006a). The feasibility and need for dimensional psychiatric diagnoses. Psychol. Med. 36, 1671–1680 10.1017/S003329170600821X 16907995

[B19] Helzer J. E. van den Brink W. Guth S. E. (2006b). Should there be both categorical and dimensional criteria for the substance use disorders in DSM-V? Addiction 101, 17–22 10.1111/j.1360-0443.2006.01587.x 16930157

[B20] Holden J. E. Finch W. H. Kelley K. (2011). A comparison of two-group classification methods. Educ. Psychol. Meas. 71, 870–901 10.1177/0013164411398357

[B21] Holden J. E. Kelley K. (2010). The effects of initially misclassified data on the effectiveness of discriminant function analysis and finite mixture modeling. Educ. Psychol. Meas. 70, 36–55 10.1177/0013164409344533

[B22] Hosmer D. W. Lemeshow S. (2000). Applied logistic Regression. Hoboken, NJ: Wiley

[B23] Hothorn T. Hornik K. Zeileis A. (2006). Unbiased recursive partitioning: a conditional inference framework. J. Comput. Graph. Stat. 15, 651–674 10.1198/106186006X133933

[B24] Huberty C. J. Olejnik S. (2006). Applied MANOVA and Discriminant Analysis. 2nd Edn Hoboken, NJ: John Wiley & Sons, Inc 10.1002/047178947X

[B25] Kamphuis J. H. Noordhof A. (2009). On categorical diagnoses in DSM-V: cutting dimensions at useful points? Psychol. Asses. 21, 294–301 10.1037/a0016697 19719342

[B26] Keogh B. K. (2005). Revisiting classification and identification. Learn. Disabil. Q. 28, 100–102 10.2307/1593603

[B27] Kim S. Olejnik S. (2005). Bias and precision of multivariate effect-size measures of association for a fixed-effect analysis of variance model. Multivariate Beh. Res. 40, 401–421 10.1207/s15327906mbr4004_1 26788828

[B28] Kleinsmith A. de Silva P. R. Bianchi-Berthouze N. (2006). Cross-cultural differences in recognizing affect from body posture. Interact. Comput. 18, 1371–1389 10.1016/j.intcom.2006.04.003

[B29] Kohavi R. (1995). The power of decision tables, in Proceedings of European Conference on Machine Learning (Heraclion).

[B30] Mardia K. V. Kent J. T. Bibby J. M. (1979). Multivariate Analysis. New York, NY: Academic Press

[B31] Maysn K. E. Henderson C. E. Greenbaum P. E. (2009). Exploring the latent structures of psychological constructs in social development using the dimensional-categorical spectrum. Soc. Dev. 19, 470–493 10.1111/j.1467-9507.2009.00573.x 24489441 PMC3905984

[B32] Meehl P. E. (1992). Factors and taxa, traits and types, differences of degree and differences in kind. J. Pers. 60, 117–174 10.1111/j.1467-6494.1992.tb00269.x

[B33] Muthen B. (2006). Should substance use disorders be considered as categorical or dimensional? Addiction 101, 6–16 10.1111/j.1360-0443.2006.01583.x 16930156

[B34] Nugent R. Dean N. Ayers E. (2010). Skill set profile clustering: the empty K-means algorithm with automatic specification of starting cluster centers in Educational Data Mining 2010: 3rd International Conference on Educational Data Mining, Proceedings, eds Baker R. S. J. d. Merceron A. Pavlik P. I. Jr. (Pittsburgh, PA), 151–160

[B35] Quinlan R. (1993). C4.5: Programs for Machine Learning. San Mateo, CA: Morgan Kaufmann Publishers

[B36] Rausch J. R. Kelley K. (2009). A comparison of linear and mixture models for discriminant analysis under non normality. Behav. Res. Methods 41, 85–98 10.3758/BRM.41.1.85 19182127

[B37] Rey M. Roth V. (2012). Copula mixture model for dependency-seeking clustering, in Proceedings of the 29th International Conference on Machine Learning (ICML) (Edinburgh).

[B38] Ruscio J. (2009). Assigning cases to groups using taxometric results. An empirical comparison of classification techniques. Assessment 16, 55–70 10.1177/1073191108320193 18607008

[B39] Schaefer J. A. Cronkite R. C. Hu K. U. (2011). Differential relationthips between continuity of care practices, engagement in continuing care, and abstinence among subgroups of patients with substance use and psychiatric disorders. J. Stud. Alcohol Drugs 72, 611–631 21683043 10.15288/jsad.2011.72.611

[B40] Schmid U. (2009). Gaussian mixture discriminant analysis for the single-cell differentiation of bacteria using micro-Raman spectroscopy. Chemometrics Intell. Lab. Syst. 96, 159–171 10.1016/j.chemolab.2009.01.008

[B41] Simonoff J. S. (1996). Smoothing Methods in Statistics. New York, NY: Springer 10.1007/978-1-4612-4026-6

[B42] Waller N. G. Jones J. A. (2009). Correlation weights in multiple regression. Psychometrika 75, 58–69

[B43] Wedekind D. Preiss B. Cohrs S. Ruether E. Huether G. Adler L. (2007). Relationship between nocturnal urinary cortisol excretion and symptom severity in subgroups of patients with depressive episodes. Neuropsychobiology 56, 119–122 10.1159/000112953 18182828

[B44] Widiger T. A. (1992). Categorical versus dimensional classification: Implications from and for research. J. Pers. Disord. 6, 287–300 10.1521/pedi.1992.6.4.287

[B45] Williams C. J. Lee S. S. Fisher R. A. Dickerman L. H. (1999). A comparison of statistical methods for prenatal screening for Down syndrome. Appl. Stochastic Models Bus. Ind. 15, 89–101 10.1002/(SICI)1526-4025(199904/06)15:2%3C89::AID-ASMB366%3E3.0.CO;2-K

[B46] Witten I. H. Frank E. (2000). Weka machine learning algorithms in java, in Data Mining: Practical Machine Learning Tools and Techniques with Java Implementations, ed Cerra D. D. (San Diego, CA: Morgan Kaufmann Publishers), 265–320

[B47] Wood S. N. (2006). Generalized Additive Models. New York, NY: Chapman and Hall

[B48] Zigler E. Phillips L. (1991). Psychiatric diagnosis: a critique. J. Abnorm. Soc. Psychol. 63, 607–618

